# Genomic analysis of serologically untypable human enteroviruses in Taiwan

**DOI:** 10.1186/s12929-019-0541-x

**Published:** 2019-07-03

**Authors:** Yeh-Sheng Chien, Shu-Ting Luo, Kuo-Chien Tsao, Yhu-Chering Huang, Wan-Yu Chung, Yu-Chieh Liao, Yi Tan, Suman R. Das, Min-Shi Lee

**Affiliations:** 1 Institute of Infectious Disease and Vaccinology, National Health Research Institutes, Zhunan, Miaoli County Taiwan; 2 Department of Life Sciences, National Central University, Taoyuan, Taiwan; 3 Department of Medical Biotechnology and Laboratory Science, College of Medicine, Chang Gung University, Guishan, Taoyuan County Taiwan; 4 Department of Pediatrics, Linkou Chang Gung Memorial Hospital, Guishan, Taoyuan County Taiwan; 5 Division of Infectious Diseases, Department of Medicine, Vanderbilt University Medical Center, Nashville, Tennessee USA; 6 Institute of Population Health Sciences, National Health Research Institutes, Zhunan, Miaoli County Taiwan; 7 National Health Research Institutes, R1-7F, 35 Keyan Road, Zhunan, Miaoli County, 350 Taiwan

**Keywords:** Enterovirus, Virus surveillance, Molecular epidemiology, Next-generation sequencing

## Abstract

**Background:**

Human enteroviruses contain over 100 serotypes. We have routinely conducted enterovirus surveillance in northern Taiwan; but about 10% of isolates could not be serotyped using traditional assays. Next-generation sequencing (NGS) is a powerful tool for genome sequencing.

**Methods:**

In this study, we established an NGS platform to conduct genome sequencing for the serologically untypable enterovirus isolates.

**Results:**

Among 130 serologically untypable isolates, 121 (93%) of them were classified into 29 serotypes using CODEHOP (COnsensus-DEgenerate Hybrid Oligonucleotide Primer)-based RT-PCR to amplify VP1 genes (VP1-CODEHOP). We further selected 52 samples for NGS and identified 59 genome sequences from 51 samples, including 8 samples containing two virus genomes. We also detected 23 genome variants (nucleotide identity < 90% compared with genome sequences in the public domain) which were potential genetic recombination, including 9 inter-serotype recombinants and 14 strains with unknown sources of recombination.

**Conclusions:**

We successfully integrated VP1-CODEHOP and NGS techniques to conduct genomic analysis of serologically untypable enteroviruses.

**Electronic supplementary material:**

The online version of this article (10.1186/s12929-019-0541-x) contains supplementary material, which is available to authorized users.

## Background

Enteroviruses (EV) are single-stranded, positive-sense RNA viruses in the Enterovirus genus of the Picornaviridae family. All enteroviruses have a similar genomic organization (7.2–8.5 kb). The capsid proteins are coded on the 5’end of the ssRNA in a section called P1 (precursor 1). The nonstructural proteins are coded on the remaining sections of the genome, which are called P2 and P3. Changes in the structural protein genes of different enterovirus species reflect phylogenetic relationships. EV cause various clinical manifestations, including cutaneous, visceral, and neurological diseases. The Enterovirus genus consists of 12 species, including Enterovirus A (EV-A, 25 serotypes), Enterovirus B (EV-B, 63 serotypes), Enterovirus C (EV-C, 23 serotypes), Enterovirus D (EV-D, 5 serotypes), Enterovirus E (EV-E, 4 serotypes), Enterovirus F (EV-F, 6 serotypes), Enterovirus G (EV-G, 11 serotypes), Enterovirus H (EV-H, 1 serotype), Enterovirus J (EV-J, 6 serotypes), Rhinovirus A (80 serotypes), Rhinovirus B (32 serotypes), and Rhinovirus C (55 serotypes) [44]. The first 4 species belong to human EV, which usually cause self-limited infections except polioviruses, EV-A71, EV-D68, and some echoviruses and coxsackieviruses [32, 38, 39]. Polioviruses have been the most important EVs for many years because they caused large outbreaks of paralytic disease before poliovirus vaccines were available.

Based on enterovirus surveillance in a medical center in northern Taiwan, there are about 10–20% of enteroviruses could not be serotyped using available monoclonal antibodies annually [41]. Those untypable enteroviruses were positive for the pan-enterovirus blend antibody but negative for all other immunofluorescent assay (IFA) antibodies, which are likely to be new serotypes or novel enteroviruses with gene mutations or recombinations [41]. Frequent recombinations and mutations in enteroviruses, which have been recognized as the main mechanisms for the observed high rate of evolution, enable EV to rapidly respond and adapt to new environmental challenges. Therefore, it is desirable to characterize these serologically untypable enteroviruses using novel molecular techniques.

The U.S. Centers for Disease Control and Prevention (CDC) has developed a primer design strategy for PCR amplification of distantly related VP1 gene sequences based on consensus-degenerate hybrid oligonucleotide primers (VP1-CODEHOP) [31]. We have employed the VP1-CODEHOP method to identify enterovirus serotypes using clinical specimens (throat swabs) [41]. Although the CODEHOP method is widely used for enterovirus typing in research labs [5, 20, 34], it is still not a routine method for enterovirus surveillance in public health labs [14]. In 2015, the World Health Organization recommend the CODEHOP method for poliovirus surveillance [1]. Recently, next-generation sequencing (NGS) technology has been applied to conduct virus genomic studies and identify novel enteroviruses [10, 33]. In this study, we first identified serologically untypable enterovirus strains through the VP1-CODEHOP method. Then enterovirus RNA were extracted for NGS to obtain the full genome sequences of enteroviruses. The enterovirus genome data were further used to identify novel enteroviruses and conduct molecular epidemiological analysis, which are critical for enterovirus surveillance and vaccine development.

## Methods

### Viruses

Chang Gung Memorial Hospital is a medical center in northern Taiwan that routinely receives clinical specimens for virus culture. The clinical specimens include respiratory (throat swabs and nasopharyngeal aspirates) and other specimens, including blood, cerebral spinal fluid, and rectal swabs. Cell culture and virus isolation were conducted according to the protocols employed commonly in clinical virology laboratories, and clinical isolates were further serotyped by immunofluorescent assay (IFA) using pan-enterovirus antibody and type-specific antibodies [41]. We obtained 130 IFA-untypable clinical isolates from Linkou Chang Gung Memorial Hospital. Human rhabdomyosarcoma (RD) cells and human lung (MRC-5) cells were used to grow enteroviruses following the standard procedures [30].

### CODEHOP method

Viral RNA was extracted using a QIAamp Mini Viral RNA Extraction Kit (Qiagen, Germany). EV VP1 gene (350 to 400 bp) was amplified as described in detail previously [31, 32]. The amplified DNA was sequenced using the ABI 3730 XL DNA Analyzer (Applied Biosystems, Foster City, CA). Nucleotide sequences of the partial VP1 gene were analyzed using the BLAST search in the GenBank database to find the enterovirus serotype with the highest identity. Alignment of the nucleotide sequences and phylogenetic analysis were conducted as described in detail previously [31, 32].

### Virus purification and concentration

The NGS technique is powerful tool for viral genome sequencing, but interference of host-cell nucleotides needs to be overcome [3, 15, 22]. To remove host-cell nucleotides, we identify a protocol to purify viruses RNA (Additional file 3). First, virus supernatant (2 ml) was treated with 0.05% formalin for 8 h for inactivation. The treated virus supernatant was filtered with 0.22 μm filter to remove cell debris. Then, virus particles were purified and concentrated using sucrose gradient ultracentrifugation. The filtered virus supernatant (1.5 mL) was loaded onto three layer of continuous sucrose buffer (PBS, 20, 30%) and centrifuged at 36,000 rpm for six hours using a Beckman SW 41-Ti rotor. We collected the virus pellet in 50 μl PBS buffer. Enteroviruses RNA were extracted after the ultracentrifugation process.

After preparing the enterovirus RNA samples, we analyzed the quality of virus RNA to eliminate contamination of ribosomal RNA. We detected the Cp value of 18 s ribosomal RNA and enterovirus RNA by real-time PCR (Additional file 1).

### Real-time PCR reaction and primers design

Enteroviruses RNA were quantified using qRT-PCR. Purified Virus RNA extraction and first strand cDNA synthesis were conducted following standard procedures [5]. EV71 viral RNA was assessed by qRT-PCR analysis with the Applied Biosystems (Thermo Fisher Scientific) Real-Time PCR system and the EV71 5’UTR primer pair. The highly conserved 5’UTR gene of the enteroviruses was chosen as the target for the synthesis of a 140-bp cDNA with primers EV509-EVF1 (5′-CCC TGA ATG CGG CTA ATC CT-3′), EV510-EVF1 (5′-CCT GAA TGC GGC TAA TCC YA-3′), and EV-R1 (5′-ATTGTCACCATAAGCAGYCR-3′).

### Next-generation sequencing (NGS)

In the pilot NGS study, we used the Illumina sequencing technology at National Yang-Ming University’s Research Center (Miseq, 2 × 250 bp reads, 40 M total reads).In NGS study two, 51 enteroviruses complete genomes were sequenced at the J. Craig Venter Institute. Illumina libraries were prepared using the Nextera DNA sample preparation kit (Illumina, San Diego, CA, USA) with half-reaction mixture volumes as described previously [39]. In order to characterize all enteroviruses serotypes, we used random primers in this study. All sequences were de novo assembled using CLC bio’s clc_ novo_assemble program (CLC Genomics Workbench 4.6.1).

### Recombination detection and phylogenetic analysis

All sequence alignments were constructed using the Muscle algorithms of the MEGA program version 4.0 (Arizona State University, AZ, USA). Potential recombinants with 1140 complete genome sequences of enteroviruses (Additional file 6) were screened using seven methods (RDP, GENECONV, MaxChi, Bootscan, Chimaera, SiScan, and 3Seq) implemented in the Recombination Detection Program version 4.46 (RDP4) [26]. The recombination events were then confirmed by constructing a phylogenetic dendrogram using the MEGA program version 4.0.

### Data access

All sequences have been submitted to the GenBank; the accession numbers are KT318494, KT353719-KT353725, and MF422531-MF422581.

## Results

### Characterization of IFA-untypable enteroviruses using the VP1-CODEHOP method

**Fig. 1:**
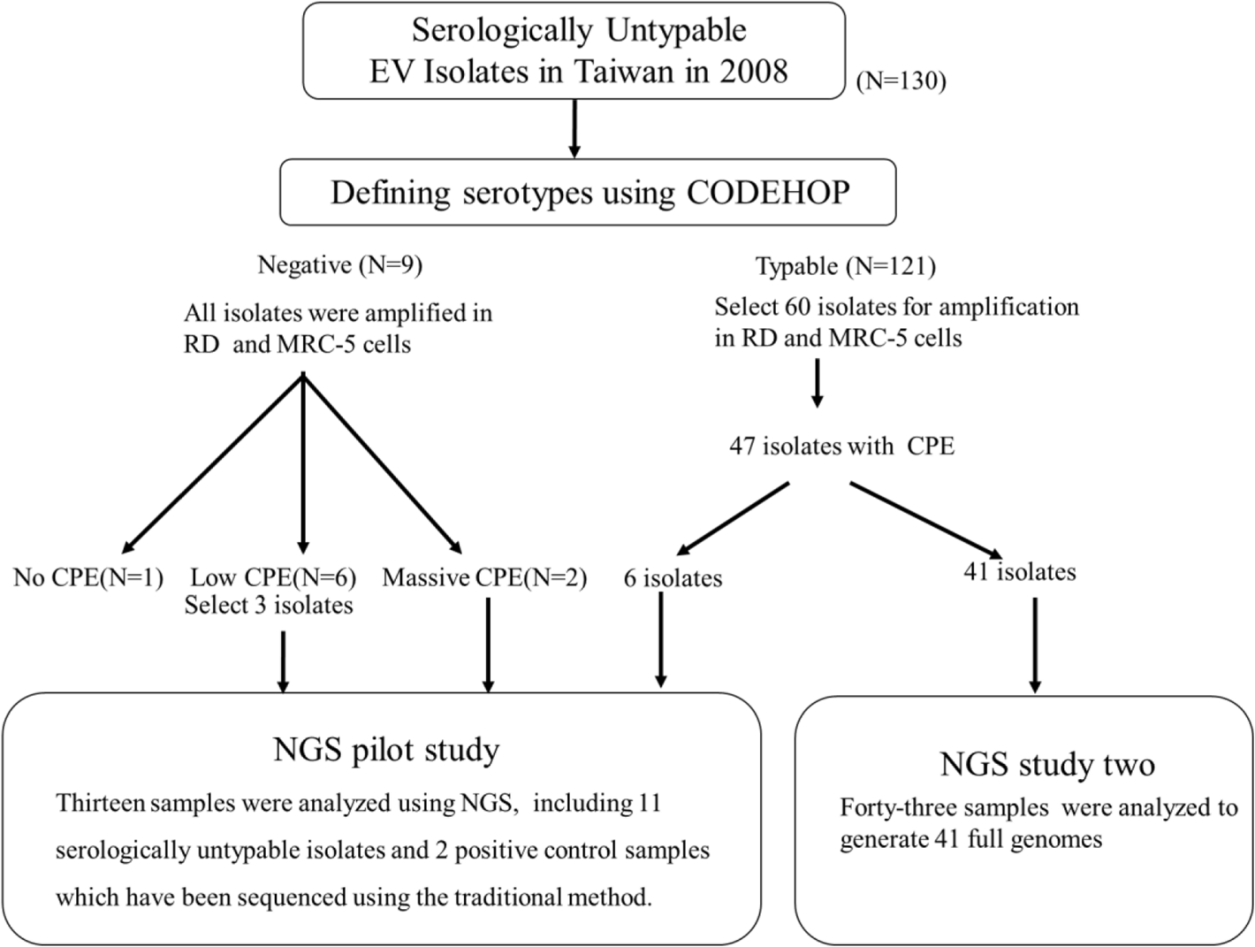
Flowchart of enterovirus genomic study

We collected 130 enterovirus isolates that could not be serotyped by IFA. Among these 130 samples, 121 were successfully identified as 19 different serotypes using the VP1-CODEHOP. The remaining 9 samples could not be identified using the VP1-CODEHOP and they were further amplified in cell cultures for genome sequencing using NGS (Fig. 1).

### Identification of untypable enterovirus by NGS

To further characterize the nine samples that could not be identified using the VP1-CODEHOP method, we first amplified these enterovirus isolates in RD and MRC-5 cells. Of these nine samples, two developed massive cytopathic effects (CPE), six developed low CPE, and one had no CPE (Fig. 1). Based on qPCR targeting virus 5’UTR, Ct for these three groups were 15–23, 34–35, and 37, respectively, which indicates that the samples without CPE had very low virus RNA concentrations and would not be suitable for NGS sequencing. In an NGS pilot study, we selected 13 samples for genome sequencing, including 2 samples with massive CPE, 3 samples with low CPE, 6 samples identified in the VP1-CODEHOP, and 2 positive control samples (EV-A71 and CV-A2) that have been sequenced with the Sanger method (Fig. 1). The distribution of read numbers for each enterovirus genome was 20,201 to 123,641 reads, and the average read number was about 80,476 reads (Additional file 5).

**Fig. 2:**
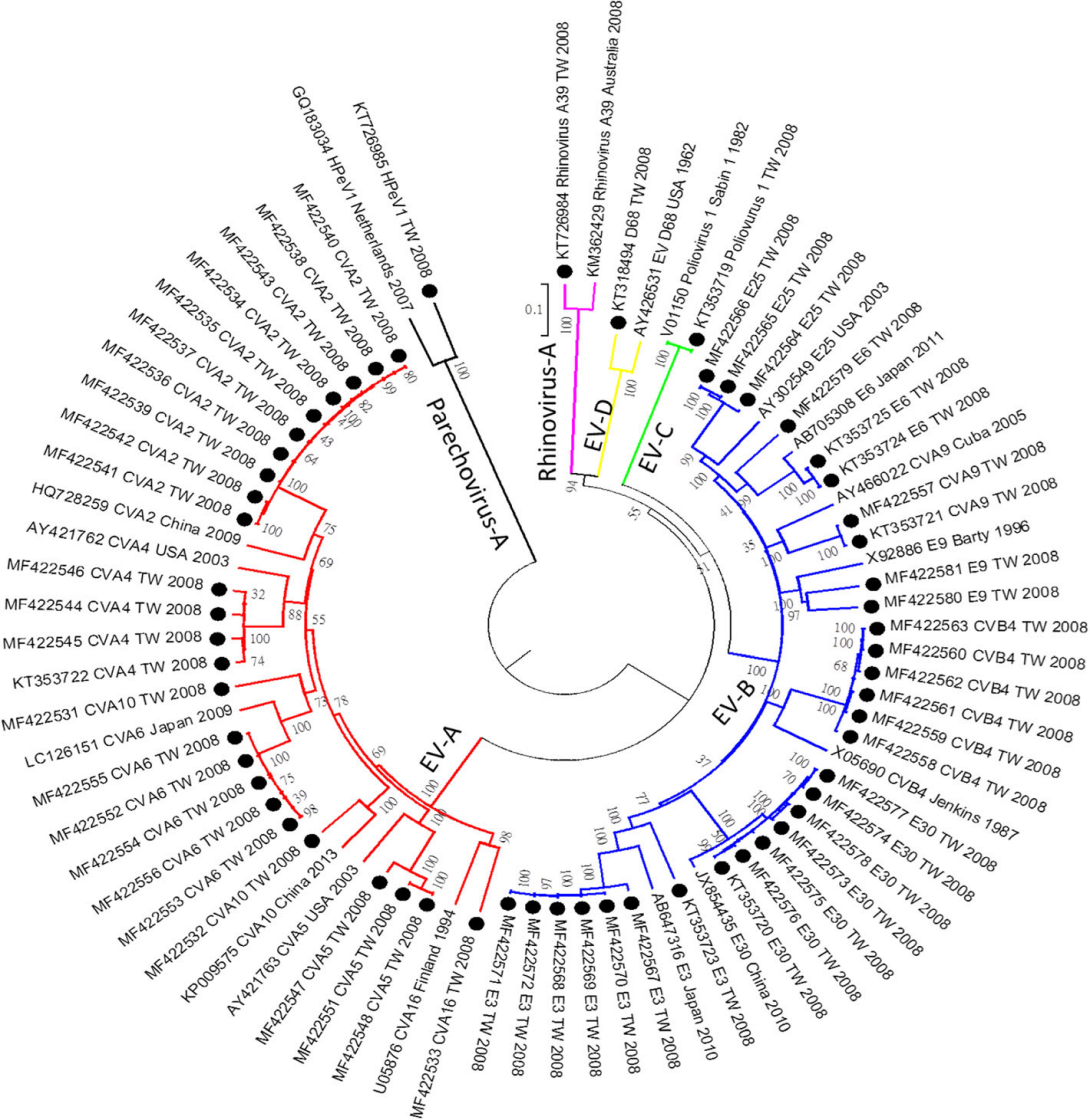
Phylogenetic analysis of serologically untypable enteroviruses detected in this study and prototype strains based on full genome sequences. The phylogenetic tree was constructed using the neighbor-joining method. Bootstrap values (> 70%) are shown as percentage derived from 1000 sampling at the nodes of the tree. Red, blue, green, yellow, and purple indicate enterovirus species **a**, **b**, **c**, and **d**, respectively

As shown in Additional file 1, we obtained 10 genome sequences from the untypable samples; and one sample (1-C2) with low CPE failed. Based on sequence alignments of the positive control samples (EV-A71 and CV-A2), identity between NGS and Sanger sequencing was 99.6% (7376/7402) and 99.9% (7306/7309), respectively. Four samples (sample ID 2-D5, 2-E6, 2-B2, and 2-B9) could not be identified using the VP1-CODEHOP, and they were sequenced as echovirus 6, echovirus 3, rhinovirus A39, and parechovirus 1 using NGS. Among the 6 samples that could be identified using the VP1-CODEHOP, all of them could also be identified and sequenced using NGS. Based on the success of the NGS pilot study, we further employed NGS to analyze other 41 samples that could be typed using the VP1-CODEHOP. Some samples were selected for NGS because their serotypes have few genome sequences available in the public domain, such as CV-A2, CV-A4, CV-A5, CV-A9, Echo 3, Echo 6, Echo 9, Echo 25, Echo 30, and rhinovirus A39 (Fig. 1 and Table 1). Among these 41 samples, 49 enterovirus genome sequences were obtained, including 8 samples with two virus genomes. Overall, we obtained 59 genome sequences from 51 cases for further analysis (Table 1). Serotypes of the genome sequences were determined by BLAST and phylogenetic analysis (Fig. 2). Demographics and clinical presentations of these 51 cases are listed in Additional file 2.

**Table 1:** Genomic characterization of serologically-untypable enteroviruses in northern Taiwan

Serotype by CODEHOP or NGS	Availability of IFA antibody	No. of isolates tested by NGS	No. of full genomes in public domain	Range of genomic identity	No. of genomic variants (identities ≤90%)
CV-A2	yes	10	14	85%	10
CV-A4	yes	4	6	97–98%	0
CV-A5	yes	5	6	96–97%	0
CV-A6	yes	5	127	98%	0
CV-A9	yes	2	25	92–99%	0
CV-A10	yes	2	65	82–86%	2
CV-A16	yes	1	105	99%	0
CV-B4	yes	6	29	96–97%	0
E3	no	7	16	85–87%	7
E6	yes	3	21	88–96%	1
E9	yes	2	12	89–90%	2
E25	no	3	7	97%	0
E30	yes	7	18	97–99%	0
PV 1	no	1	258	99%	0
EV-D68	no	1	388	97%	0
RV-A39^*^	no	1	3	93%	0
HPeV 1^*^	no	1	38	85%	1
Total		59			23

^*^ Sample cannot be identified by CODEHOP method

### Recombination detection

**Fig. 3:**
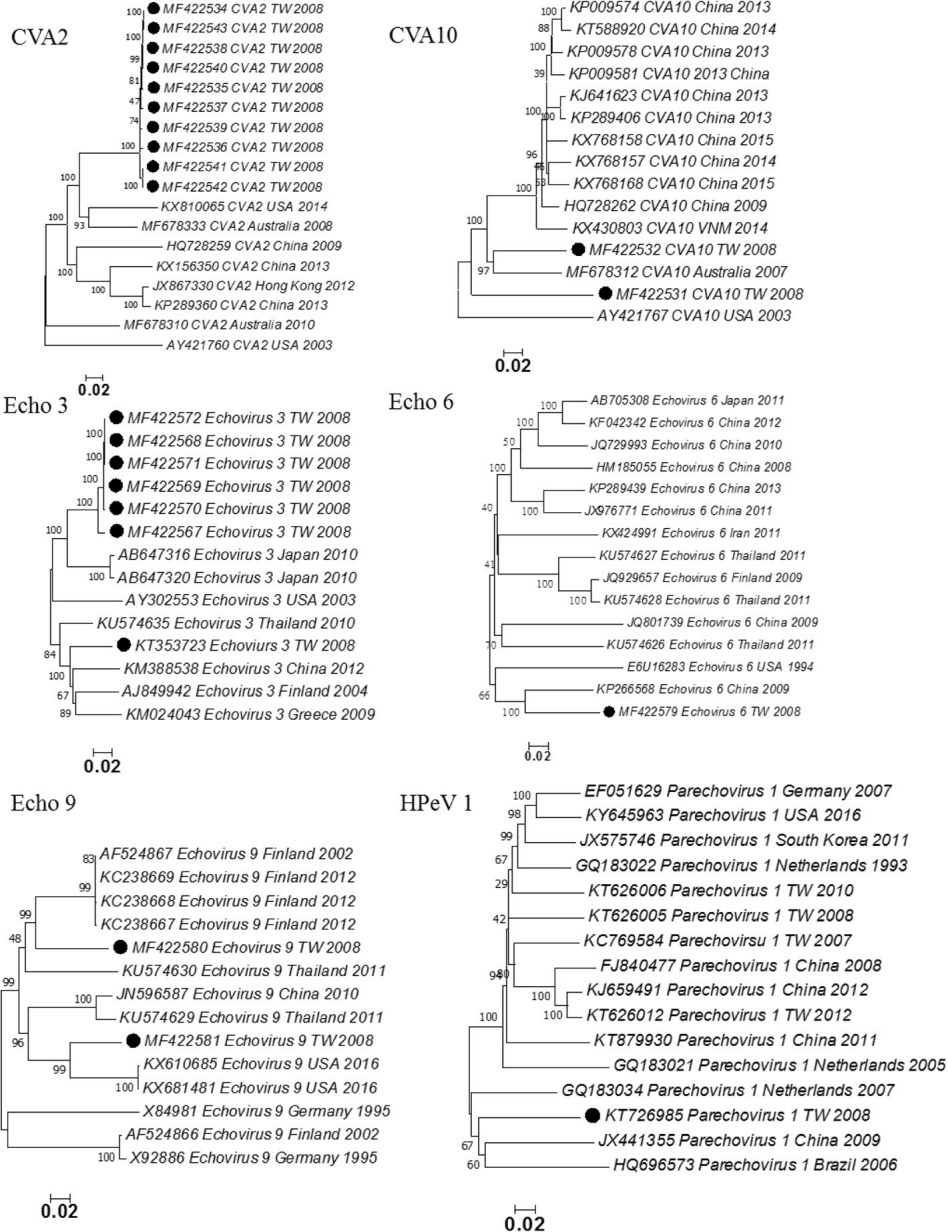
Phylogenetic analysis of six enterovirus serotypes with genomic variants detected. Black dot indicates isolates sequenced in this study. The phylogenetic tree was constructed using the neighbor-joining method. Bootstrap values (> 70%) are shown as percentage derived from 1000 sampling at the nodes of the tree

**Fig. 4:**
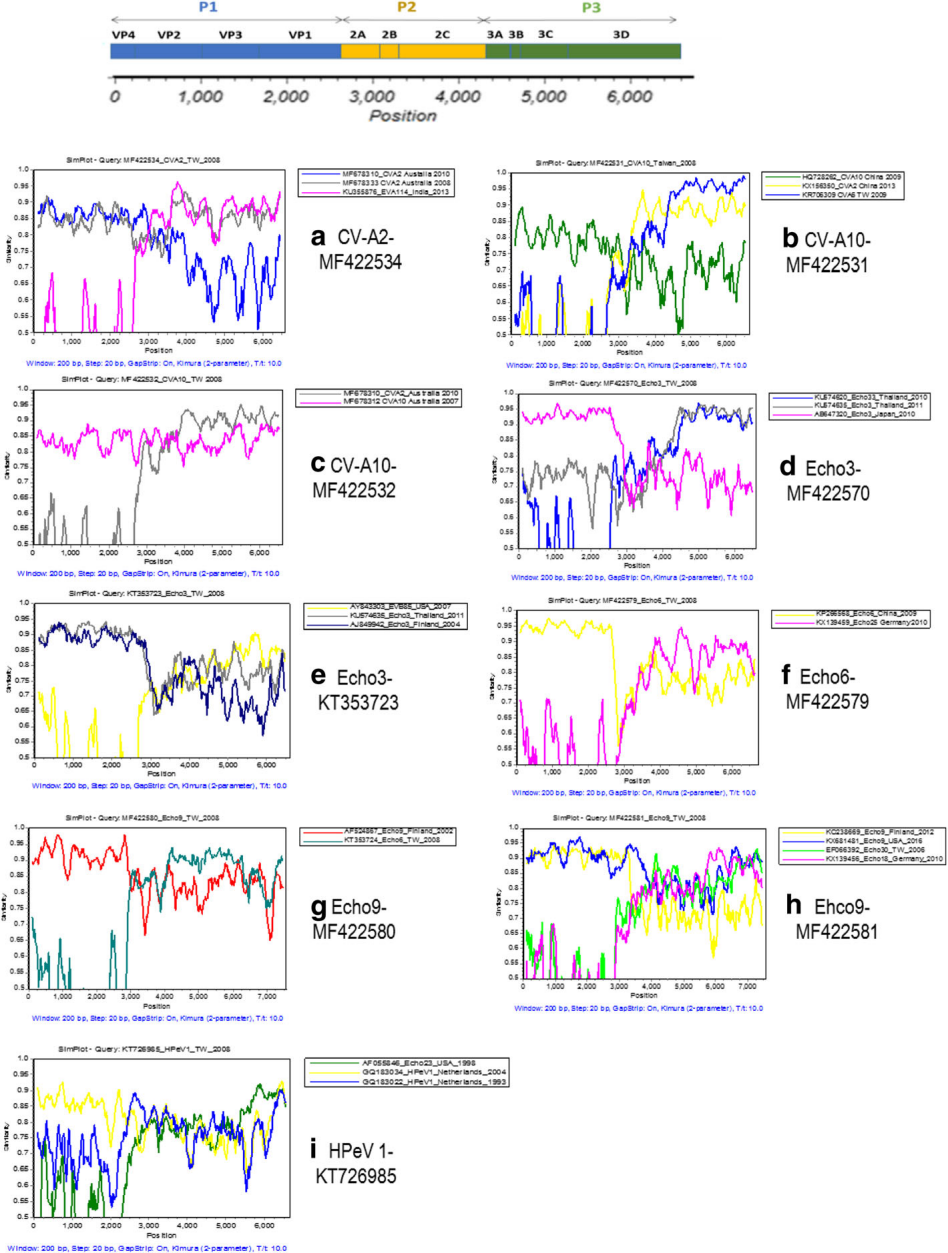
Recombination analysis of nine genomic variants using SimPlot. (**a**) CV-A2-MF422534, (**b**) CV-A10-MF422531, (**c**) CV-A10-MF422532, (**d**) Echo3-MF422570, (**e**) Echo3-KT353723, (**f**) Echo6-MF422579, (**g**) Echo9-MF422580, (**h**) Ehco9-MF422581, and (**i**) HPeV 1-KT726985. Similarity plots comparing query strain with the reference strains selected using BLAST analysis (Table 2). The analyses were conducted via Simplot v3.5.1 using a sliding window of 200 nucleotides, moving in steps of 20 nucleotides

Based on phylogenetic analysis, serotypes of the 59 genomes could be classified into 17 enterovirus serotypes (Fig. 2). There is no official definition of genome variants. To identify recombinant viruses, we selected genome variant of 90% identity as screening standards. Among them, 23 genomes had low genetic identity (< 90%) compared with sequence data in the public domain and they are considered as genomic variants which may be derived from genetic recombination. These 23 genomes were classified into six serotypes (CV-A2, CV-A10, HPeV 1, Echo 3, Echo 6, and Echo 9). Among them, three serotypes (CV-A10, Echo 3, and Echo 9) have two genome groups based on phylogenetic analysis (Fig. 3). Based on analysis using the RDP program, we detected only one potential recombination event. Therefore, we tried to identify recombination events by dividing the 23 genomes into P1, P2, and P3 segments for BLAST analysis (Table 2). Potential breakpoints of the nine genomic groups were further identified using SimPlot (Fig. 4). The 10 CV-A2 variants clustered together (Fig. 3), but their recombination events could not be identified (Table 2) (Fig. 4a). Based on BLAST analysis of the VP1 genes, the CV-A2 variants are closest to the CV-A2 viruses isolated in Japan in 2003 (Table 2), which is consistent with phylogenetic analysis of the VP1 genes (data not shown). All CV-A2 cases were mild infections without neurological complications (Additional file 2).

**Table 2:** Top sequence identity identified in BLAST analysis of 29 genomic variants

Serotype (No.)	Virus ID	P1 region	Identity	P2 region	Identity	P3 region	Identity	Break point
CVA2 (10)	MF422534-MF422543	CVA2 Australia 2010 MF678310	88%	EVA114 India 2013 KU355876	87%	EVA114 India 2013 KU355876	89%	unknown
		(CVA2 Japan2003 AB162722)^a^	(93%)^a^	CVA2 Australia 2008 MF678333	86%	CVA2 Australia 2008 MF678333	88%	
CVA10 (2)	MF422531	CVA10 Australia 2007 MF678312	86%	CVA2 China 2013 KX156350	85%	CVA6 TW 2007 KR706309	96%	P1-P2,P2-P3
		(CVA10 China 2008 GQ214174)^a^	(97%)^a^					
	MF422532	CVA10 China 2013 KP009581	85%	CVA2 Australia 2010 MF678310	86%	CVA2 Australia 2010 MF678310	91%	unknown
		(CVA10 Australia 2007 MF678312)^a^	(86%)^a^					
Echo3 (7)	MF422567-MF422572	Echo3 Japan 2010 AB647320	94%	Echo33 Thailand 2011 KU574620	83%	Echo3 Thailand 2010 KU574635	94%	P1-P2,P2-P3
	KT353723	Echo3 Thailand 2010 KU574635	92%	Echo3 Finland 2006 AJ849942	82%	EVB85 USA 2007 AY843303	86%	unknown
Echo6 (1)	MF422579	Echo6 China 2009 KP266568	95%	Echo25 Germany 2010 KX139459	82%	Echo25 Germany 2010 KX139459	88%	P1-P2
Echo9 (2)	MF422580	Echo 9 Finland 2002 AF524867	92%	Echo6 TW 2008 KT353724	88%	Echo6 TW 2008 KT353724	89%	P1-P2
	MF422581	Echo9 USA 2016 KX681481	92%	Echo 9 USA 2016 KX681481	88%	Echo 18 Germany 2010 KX139456	88%	unknown
		(Echo9 Finland 2012 KC238669)^b^	(92%)^b^	(Echo 9 Finland 2012 KC238669)^b^	(83%)^b^	(Echo 9 Finland 2012 KC238669)^b^	(79%)^b^	
		(Echo 30 TW 2006 EF066392)^b^	(69%)^b^	(Echo 30 TW 2006 EF066392)^b^	(83%)^b^	(Echo 30 TW 2006 EF066392)^b^	(86%)^b^	
HPeV1 (1)	KT726985	HPeV 1 Netherlands 2007 GQ183034	88%	HPeV1 Netherlands 1993 GQ183022	85%	HPeV 1 China 2009 JX441355	86%	unknown
		(HPeV 1 Australia 2010 MG712784)^a^	(87%)^a^					

^a^Blast result of VP1 gene; ^b^Strain selected by RDP 4.0 program

The two CV-A10 variants could be classified into two genomic groups (Fig. 3). The first CV-A10 variant (strain MF422531) is likely a triple recombinant of CV-A10, CV-A2, and CV-A6 (Fig. 4b); and the recombination event of the second CV-A10 variant could not be identified (Fig. 4c). The two CV-A10 cases were mild infections. The seven Echo3 variants could be classified into two groups (Fig. 3). The first Echo3 group has six genomic variants, which are likely recombinants of Echo3 and Echo33 (Fig. 4d); but the recombination event of the second Echo3 group could not be identified (Fig. 4e). One case in the first genome group develops bacteremia and neurological complications (case 30, Additional file 2). The only Echo6 variant is likely a recombinant of Echo6 and Echo25 (Fig. 4f), and it caused mild infection.

The two Echo9 variants could be classified into two groups (Fig. 3). The first group (MFF422580) is likely a recombinant of Echo9 and Echo6 (Fig. 4g); and the recombination event of the second group (MFF422581) could not be identified (Fig. 4h). Of these two Echo9 cases, the first (MFF422580) causes mild infection while the other (MFF422581) causes meningitis. Interestingly, the RDP program predicted that the Echo9 strain MFF422581 is likely a recombinant of Echo9 Finland 2012 and Echo30 Taiwan 2006 (Additional file 4), but the recombination event could not be confirmed in the BLAST and SimPlot analysis (Table 2) (Fig. 4h). The recombination event of the HPeV1 variant could not be identified (Table 2) (Fig. 4i). Based on BLAST analysis of the VP1 genes, the HPeV1 variant is close to the HPeV1 that circulated in Australia 2010 (Table 2). This HPeV1 causes a mild infection. Overall, 9 of the 23 genomic variants were identified as inter-serotype recombinants, and recombination events of the other 14 genomic variants could not be identified due to lack of comprehensive genome sequences (Table 2).

### Virus co-infection and clinical presentation

Interestingly, we found eight patients who were co-infected with two enterovirus serotypes (cases 4, 16, 18, 22, 23, 28, 31, and 32) (Additional file 2) by using NGS. Co-infections could not be easily detected by the CODEHOP method because the dominant serotype will overwhelmingly surpass the minor serotype during the gene-amplification process.

Among these eight cases, three developed neurological complications (cases 4, 31, and 32) and one had pneumonia (case 23). Among the other 43 cases with a single enterovirus infection, only 4 (cases 33, 37, 48, and 49) had neurological complications. Overall, enterovirus co-infections were more likely to cause neurological complications than single enterovirus infections (*p* = 0.015, Fisher’s Exact Test).

## Discussion

Molecular techniques have gained increasing attention for virus surveillance and the clinical management of infectious diseases. Traditional methods for virus surveillance, including virus isolation and IFA tests, require 5–14 days to complete detection and serotyping. In contrast, the VP1-CODEHOP test can finish detection and serotyping within 48 h [6]. In the current study, serotypes of nine virus samples could not be identified using the VP1-CODEHOP. Therefore, we further employed NGS to characterize virus samples that could not be identified using the VP1-CODEHOP. The NGS technique can obtain full genome sequences without the requirement of designing specific primers, but it requires collecting purified virus nucleotide [3, 37, 40]. Therefore, we used sucrose-cushioned ultracentrifugation to purify the virus nucleotide. Among 52 virus samples, only one sample with low CPE could not be sequenced using this platform. Overall, we successfully integrated VP1-CODEHOP and NGS techniques to improve enterovirus surveillance.

Recombination plays a crucial role in viral evolution and adaptation by repairing deleterious mutations in genomes, thus rescuing viral genes from low-fitness parents. Two viruses can exchange genetic material only in the case of co-infection of the same host cell. Two models of recombination have been proposed for enteroviruses: the template-switch model and the breaking-joining model [11, 23, 24]. In this study, we obtained 59 complete genome sequences for molecular epidemiological study. Among them, 23 genome variants were detected, and recombination events of nine genome variants were identified (1 CV-A10 strain, 6 Echo3 strains, 1 Echo6 strain, and 1 Echo9 strain).

Due to a lack of reference genomes, the recombination events of the remaining 14 genome variants could not be confirmed. Enteroviruses are RNA viruses and have high mutation rates and frequent recombination [23, 25, 29]. Therefore, it is desirable to generate more genome sequence data to understand the evolution of enteroviruses.

Many studies have revealed that recombination is a frequent phenomenon among enteroviruses. Interestingly, recombination events have been observed more frequently among members of the same species and have been detected mostly in strains from species B [23]. In our study, we also detected more gene recombination in species B viruses (Echo3, Echo6, and Echo9).

In our study, we detected 10 CV-A2 genome variants, which phylogenetically clustered together; but their recombination events could not be identified. Currently, there are about 14 CV-A2 genome sequences in the public domain, with only one strain collected before 2008. Therefore, it is hard to elucidate the evolution of the CV-A2 strains isolated in our study. Moreover, four CV-A2 viruses were isolated from an AFP patient in India from 2007 to 2009 [35] and variant CV-A2 caused four complicated cases in Hong Kong in 2012 [43]. The CV-A2 cases detected in our study resulted in only mild infections, but CV-A2 viruses have the potential to cause severe infections and need to be monitored intensively.

In this study, we found a genome variant of CV-A10 (MF422531) that might be a triple recombinant of CV-A10, CV-A2, and CV-A6, with the breakpoints occurring in P1/P2 and P2/P3 junctions. The recombination event of the other CV-A10 variant (MF422532) could not be identified. In a previous study, Hu et al. found that CVA10 isolated in China in 2009 is a recombinant between CV-A10 and EV-A, but the source of its P2 gene could not be confirmed [16]. Currently, about 65 CV-A10 genome sequences are in the public domain, only one of which was collected before 2008. Therefore, it is desirable to sequence more CV-A10 strains isolated before 2008.

EV-B is the most abundant species of enterovirus (63 serotypes), and intra species recombination of EV-B enterovirus has occurred frequently in the last decade [23, 44]. In our study, we also found that 3 of 6 serotypes (50%) of EV-B enteroviruses were detected with genome recombination, which is higher than that of EV-A (2/7) (Table 3). The only Echo6 variant is likely a recombinant of Echo6 and Echo25 and it causes mild infection (case 35, Additional file 2). Interestingly, the Echo6 genomic variants are not phylogenetically close to the other two Echo6 cases, which developed severe neurological complications (cases 48 and 49, Additional file 2). It is well documented that Echo6 causes seasonal epidemics of aseptic meningitis [2]. There are about 21 Echo6 genome sequences in the public domain, and only three of them were collected before 2008. Therefore, it is necessary to actively monitor the Echo6 virus and sequence more Echo6 strains isolated before 2008.

In our study, an Echo9 variant (MF422581) was found to be a recombinant of Echo9 and Echo30 using the RDP program; but the recombination event could not be confirmed using BLAST and Simplot analysis. In general, RDP is an automatic program for screening recombination events, and BLAST and Simplot analyses are more reliable. Therefore, the recombination events detected using RDP need to be verified using BLAST and Simplot analyses. There are about 12 Echo9 genome sequences in the public domain, and it is necessary to sequence more Echo9 strains. One Echo9 (MF422580) genomic variant causes mild infection and the other Echo9 (MF22581) causes aseptic meningitis, which is consistent with other studies that found Echo9 strains frequently cause aseptic meningitis in Asia [17, 45].

Human parechovirus (HPeV) were first detected in 1956 and classified into 19 serotypes. HPeV primarily causes sepsis and central nervous system diseases in infants, and still has other unproven clinical manifestations [36]. HPeV is not yet included for serotyping using IFA in the Taiwan Virology Reference Laboratory Network, but they have been detected using molecular techniques in Taiwan since 2007 [18]. It has been documented that HPeV culture is still limited due to the low induction of CPE, and more sensitive VP1 primers are required for different HPeV genotypes [4, 42]. In our study, the HPeV strain could not be detected using the CODEHOP method but could be identified using NGS technology without the requirement of designing specific primers. Interestingly, the HPeV1 strain (KT726985) in our study was phylogenetically classified into Clade A — in contrast to other Taiwan HPeV1 strains, which belong to the Clade B circulating in 2007–2012 [12, 18]. This HPeV 1 (KT726985) strain is a genomic variant but its source could not be identified due to limited genome sequence and high variability.

Life-threatening outbreaks of EV-D68 emerged in 2014 in North United States [9] and gradually spread to multiple countries [7, 19, 27]. EV-D68 infections in children usually manifest with respiratory symptoms and may cause neurological complications. It is necessary to strengthen EV-D68 detection globally [39]. Virus isolation from cell culture has been used for many years for the detection of enteroviruses. RT-PCR is generally more sensitive and more rapid than virus isolation [5, 6], but not all laboratories are equipped to test for enteroviruses. Moreover, many available laboratory methods for the detection of EVs cannot distinguish between enteroviruses and rhinoviruses, and provide no information on serotypes [21]. Some of the severe rhinovirus infections previously described during the 2009–2014 period were actually EV-D68 [28]. In the current study, we detected one EV-D68 strain using the CODEHOP method. Recently, the Taiwan Centers for Disease Control further detected EV-D68 from acute flaccid paralysis patients using the CODEHOP method. Therefore, the CODEHOP method could be widely used for detection of EV-D68.

Previous studies found that enterovirus co-infection may cause more severe symptoms [8, 13]. Enterovirus co-infections are seldom detected using the traditional IFA and RT-PCR methods, because these methods are more likely to detect the predominant strain in a clinical specimen. We detected eight co-infection events using the NGS method in this study. Therefore, VP1-CODEHOP and NGS could be used together for genomic analysis of serologically untypable enteroviruses. Both of them should be integrated into enterovirus surveillance to help clinical management and identification of novel enteroviruses.

## Conclusion

Cost is a critical issue for the widespread use of NGS. In the pilot study, we first concentrated enterovirus with ultracentrifugation. Then we generated 3–10 million reads for each concentrated enterovirus sample and the NGS sample 1–10 genome sequence were obtained through de novo assembly (Additional file 2). The distribution of read depth for each enterovirus genome was 20,201 to 123,641 reads; and the average read depth was about 80,476, which indicates that compression of sequencing reads is a possible way to reduce the cost of NGS sequencing (Additional file 5). In the NGS study two, we generated 0.3–1.5 million reads for each concentrated enterovirus sample to reduce sequencing cost. Based on our results, we believe that 0.5 million reads of raw data would suffice for NGS sequencing of each concentrated enterovirus sample; this could further reduce the cost of sequencing. In the pilot study, we generated 3–10 megabyte reads through a contract research organization; this cost about US$500 per sample. In the NGS study two, we collaborated with the J. Craig Venter Institute and generated 0.3–1.5 megabyte reads for each sample. It is hard to precisely calculate the cost of library construction and genome sequencing in the NGS study two.

In conclusion, we successfully integrated VP1-CODEHOP and NGS techniques to conduct genomic analysis of serologically untypable enteroviruses, which could not only improve enterovirus surveillance but also provide genome sequences for evolution research.

## Additional files

Additional file 1: Analysis of enterovirus genomes sequenced using NGS in a pilot study. (DOCX 21 kb)

Additional file 2: Characteristics of enterovirus patients characterized using NGS in Taiwan, 2008. (DOCX 23 kb)

Additional file 3: Flowchart of the next-generation sequencing (NGS) method. (PDF 85 kb)

Additional file 4: Recombination analysis of MF422581_E9_TW_2008 using the RDP 4.0 program. The analyses were conducted via RDP 4.0 using the manual Bootscan method. Windows200 nt, step 20 nt. (PDF 194 kb)

Additional file 5: Sequencing coverage of each NGS sample. (XLSX 23 kb)

Additional file 6: Contributions of all enterovirus reference strain serotype. (XLSX 13 kb)

## Data Availability

All data generated or analyzed during this study are included in this published article [and its supplementary information files].
