# Supplementary material for: Genomic analysis of serologically untypable human enteroviruses in Taiwan
Source: J Biomed Sci. 2019 Jul 3;26:49. doi: 10.1186/s12929-019-0541-x (PMC6607526; doi:10.1186/s12929-019-0541-x)
Supplement: Supplementary file 1 — Analysis of enterovirus genomes sequenced using NGS in a pilot study. (DOCX 21 kb) [file 12929_2019_541_MOESM1_ESM.docx]

Additional file 1. Analysis of enterovirus genomes sequenced using NGS in a pilot study

| **NGS sample** | **Sample**  **ID** | **Accession Number** | **Serotype**  **(CODEHOP)** | **Serotype**  **(NGS)** | **Availability of IFA antibody** | **Top Identity** | **Top Identity strain** | **enterovirus reads** | **Total reads** |
| --- | --- | --- | --- | --- | --- | --- | --- | --- | --- |
| 1 | EV71* |  | EV-A71 | EV-A71 | Yes | 99.6% | KF974783 | 3,201,719 | 3,251,510 |
| 2 | CVA2* |  | CV-A2 | CV-A2 | Yes | 99.9% | JX867333 | 3,241,533 | 3,300,420 |
| 3 | 1-I8 | KT318494 | EV-D68 | EV-D68 | No | 97% | KM892501 | 3,937,405 | 4,112,564 |
| 4 | 1-B2 | KT353719 | poliovirus type 1(Sabin strain) | poliovirus type 1(Sabin strain) | Yes | 99% | AY184219 | 2,188,581 | 2,438,394 |
| 5 | 1-E9 | KT353722 | CV-A4 | CV-A4 | Yes | 98% | JX867333 | 3,863,575 | 3,931,444 |
| 6 | 1-B4 | KT353720 | Echo 30 | Echo 30 | Yes | 97% | JN704615 | 4,556,382 | 4,668,740 |
| 7 | 1-D7 | KT353721 | CV-A9 | CV-A9 | Yes | 92% | KM890278 | 3,229,215 | 3,300,286 |
| 8 | 2-C2 | KT353724 | Echo 6 | Echo 6 | Yes | 96% | AB705311 | 3,842,230 | 4,034,610 |
| 9 | 2-D5 | KT353725 | Untypable | Echo 6 | Yes | 96% | AB705311 | 2,864,105 | 2,960,912 |
| 10 | 2-E6 | KT353723 | Untypable | Echo 3 | No | 85% | AJ849942 | 3,373,635 | 3,435,102 |
| 11 | 2-B2 | KT726984 | Untypable | Rhinovirus A39 | No | 93% | AY751783 | 1,686,754 | 10,012,526 |
| 12 | 2-B9 | KT726985 | Untypable | Parechovirus 1 | No | 85% | GQ183034 | 720,779 | 8,748,522 |
| 13 | 1-C2 |  | Negative | Failed | - | - | - | 0 | 11,251,172 |

*: positive control sample, have been sequenced with the Sanger method
