# Supplementary material for: Genomic analysis of serologically untypable human enteroviruses in Taiwan
Source: J Biomed Sci. 2019 Jul 3;26:49. doi: 10.1186/s12929-019-0541-x (PMC6607526; doi:10.1186/s12929-019-0541-x)
Supplement: Supplementary file 2 — Characteristics of enterovirus patients characterized using NGS in Taiwan, 2008. (DOCX 23 kb) [file 12929_2019_541_MOESM2_ESM.docx]

Additional file 2. Characteristics of enterovirus patients characterized using NGS in Taiwan, 2008.

| Case | Age | Symptoms | Hospitalization | Serotype | Accession No. | LAB ID |
| --- | --- | --- | --- | --- | --- | --- |
| 1 | 3 | herpangina | none | Coxsackievirus A10 | MF422531 | 61216 |
| 2 | 3 | herpangina | 3 days | Coxsackievirus A10 | MF422532 | 61217 |
| 3 | 4 | herpangina | none | Coxsackievirus A16 | MF422533 | 61218 |
| 4* | 5 | febrile convulsions/sepsis | none | Coxsackievirus A2 | MF422534 | 61219 |
|  |  |  |  | Echovirus 30 | MF422577 | 63037 |
| 5 | 1 | herpangina | none | Coxsackievirus A2 | MF422535 | 61220 |
| 6 | 1 | herpangina | none | Coxsackievirus A2 | MF422536 | 61221 |
| 7 | 1 | herpangina | none | Coxsackievirus A2 | MF422537 | 61222 |
| 8 | 3 | herpangina/myoclonic jerk | 2 days | Coxsackievirus A2 | MF422538 | 61223 |
| 9 | 2 | acute tonsillitis | none | Coxsackievirus A2 | MF422539 | 61224 |
| 10 | 3 | Herpangina/myoclonic jerk | 2 days | Coxsackievirus A2 | MF422540 | 61225 |
| 11 | 6 | herpangina | none | Coxsackievirus A2 | MF422541 | 61226 |
| 12 | 2 | herpangina | none | Coxsackievirus A4 | MF422544 | 61227 |
| 13 | 1 | herpangina | none | Coxsackievirus A4 | MF422545 | 61228 |
| 14 | 6 | pneumonia | none | Coxsackievirus A4 | MF422546 | 61229 |
| 15 | 1 | Unknown | none | Coxsackievirus A5 | MF422547 | 61230 |
| 16* | 1 | herpangina | none | Coxsackievirus A5 | MF422548 | 61231 |
|  |  |  |  | Echovirus 3 | MF422571 | 63038 |
| 17 | 2 | acute tonsillitis | 9 days | Coxsackievirus A6 | MF422552 | 61234 |
| 18* | 1 | herpangina | 2 days | Coxsackievirus A6 | MF422553 | 61235 |
|  |  |  |  | Echovirus 30 | MF422578 | 63039 |
| 19 | 1 | herpangina | 2 days | Coxsackievirus A6 | MF422554 | 61236 |
| 20 | 4 | herpangina/pneumonia | none | Coxsackievirus A6 | MF422555 | 61237 |
| 21 | 1 | herpangina | none | Coxsackievirus A9 | MF422557 | 61238 |
| 22* | 5 | acute tonsillitis | 4 days | Coxsackievirus B4 | MF422558 | 61239 |
|  |  |  |  | Echovirus 3 | MF422572 | 63040 |
| 23* | 7 | pneumonia | 3 days | Coxsackievirus B4 | MF422559 | 61240 |
|  |  |  |  | Coxsackievirus A2 | MF422542 | 63041 |
| 24 | 2 month | respiratory virus infection | none | Echovirus 25 | MF422564 | 61241 |
| 25 | 1 | skin rash suspect virus exanthem | 4 days | Echovirus 25 | MF422565 | 61242 |
| 26 | 1 | Respiratory virus infection | none | Echovirus 25 | MF422566 | 61243 |
| 27 | 1 | acute gastroenteritis /fever/virus infection | 2 days | Echovirus 3 | MF422567 | 61244 |
| 28* | 1 | fever | 3 days | Echovirus 3 | MF422568 | 61245 |
|  |  |  |  | Coxsackievirus A5 | MF422551 | 63042 |
| 29 | 5 | acute tonsillitis | 3 days | Echovirus 3 | MF422569 | 61246 |
| 30 | 3 | exudative tonsillitis /bacteremia with CNS | 6 days | Echovirus 3 | MF422570 | 61247 |
| 31* | 7 | aseptic meningitis /upper respiratory tract infection | 6 days | Echovirus 30 | MF422573 | 61248 |
|  |  |  |  | Coxsackievirus A6 | MF422556 | 63043 |
| 32* | 13 | aseptic meningitis/ Sinusitis | none | Echovirus 30 | MF422574 | 61249 |
|  |  |  |  | Coxsackievirus A2 | MF422543 | 63044 |
| 33 | 5 | acute pharyngitis/suspect aseptic meningitis | 2 days | Echovirus 30 | MF422575 | 61250 |
| 34 | 7 | vomiting/throat injected | none | Echovirus 30 | MF422576 | 61251 |
| 35 | 9 | headache/fever | none | Echovirus 6 | MF422579 | 61252 |
| 36 | 2 | fever/favor viral syndrome | 3 days | Echovirus 9 | MF422580 | 61253 |
| 37 | 7 | aseptic meningitis | 7 days | Echovirus 9 | MF422581 | 61254 |
| 38 | 4 | acute pharyngitis | 4 days | Coxsackievirus B4 | MF422560 | 61255 |
| 39 | 4 | HFMD | 3 days | Coxsackievirus B4 | MF422561 | 61256 |
| 40 | 5 | acute tonsillitis/fever | 5 days | Coxsackievirus B4 | MF422562 | 61257 |
| 41 | 3 | fever, pharyngeal vesicles or ulcer, herpangia | none | Coxsackievirus B4 | MF422563 | 61258 |
| 42^#^ | 4 | Jaundice /mouth and throat ulcer /emergency liver transplantation | 51 days | Enterovirus 68 | KT318494 | 755 |
| 43^#^ | 1 | fever | none | Poliovirus 1 | KT353719 | 1980 |
| 44^#^ | 1 | HFMD | 6 days | Echovirus 30 | KT353720 | 1582 |
| 45^#^ | 7 | herpangina | none | Coxsackievirus A9 | KT353721 | 2558 |
| 46^#^ | 4 | fever/ febrile | none | Coxsackievirus A4 | KT353722 | 2811 |
| 47^#^ | 12 | fever, myalgia, sore throat | none | Echovirus 3 | KT353723 | 71190 |
| 48^#^ | 7 | aseptic meningitis | 6 days | Echovirus 6 | KT353724 | 940 |
| 49^#^ | 7 | aseptic meningitis | none | Echovirus 6 | KT353725 | 939 |
| 50^#^ | 2 | cough/asthma | none | Rhinovirus A 39 | KT726984 | 427 |
| 51^#^ | 1 | fever/oral thrush | 6 days | Parechovirus 1 | KT726985 | 1245 |

* Case of enteroviruses co-infection

# Case of enteroviruses in the pilot study
