# Supplementary figures and images for: Genomic analysis of serologically untypable human enteroviruses in Taiwan
Source: J Biomed Sci. 2019 Jul 3;26:49. doi: 10.1186/s12929-019-0541-x (PMC6607526; doi:10.1186/s12929-019-0541-x)

Additional file 3

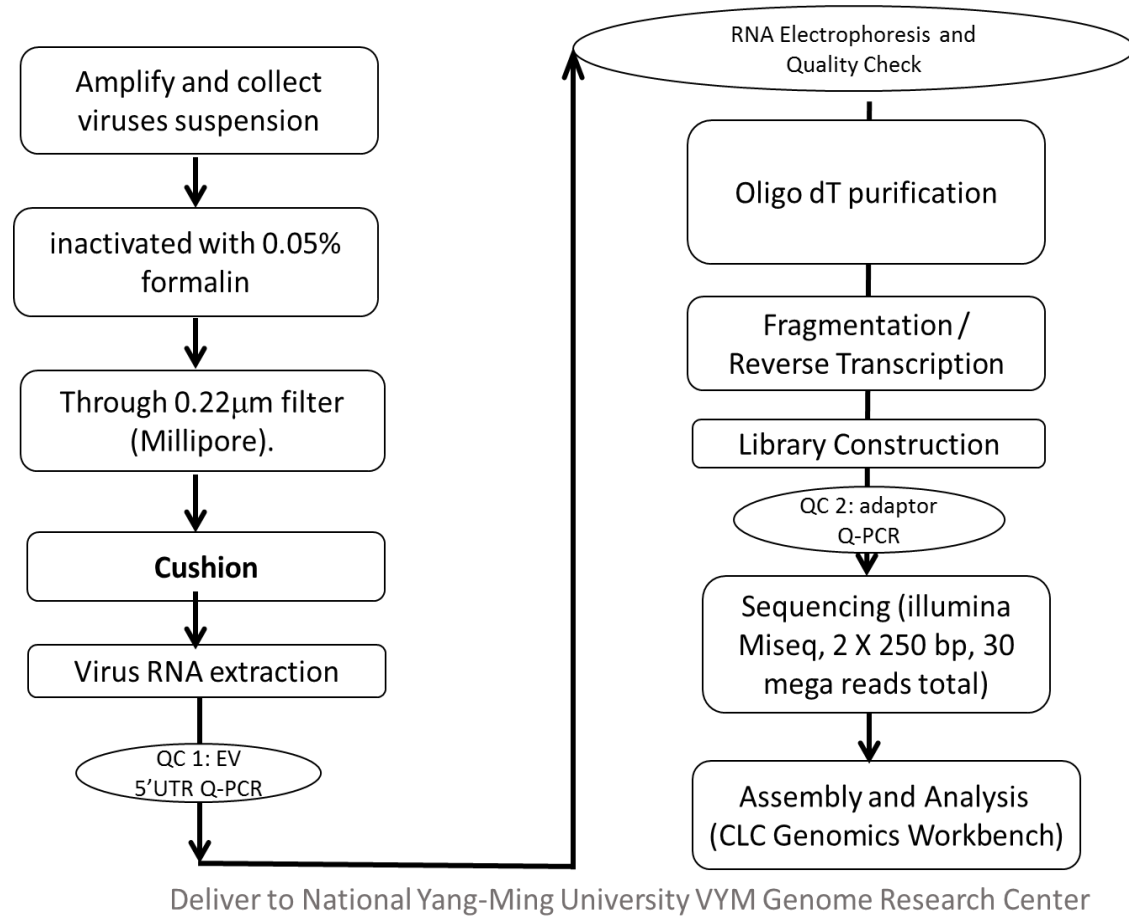

Supplement: Supplementary file 3 — Flowchart of the next-generation sequencing (NGS) method. (PDF 85 kb) [file 12929_2019_541_MOESM3_ESM.pdf]
