# Supplementary material for: Genomic analysis of serologically untypable human enteroviruses in Taiwan
Source: J Biomed Sci. 2019 Jul 3;26:49. doi: 10.1186/s12929-019-0541-x (PMC6607526; doi:10.1186/s12929-019-0541-x)

Additional file 4 .

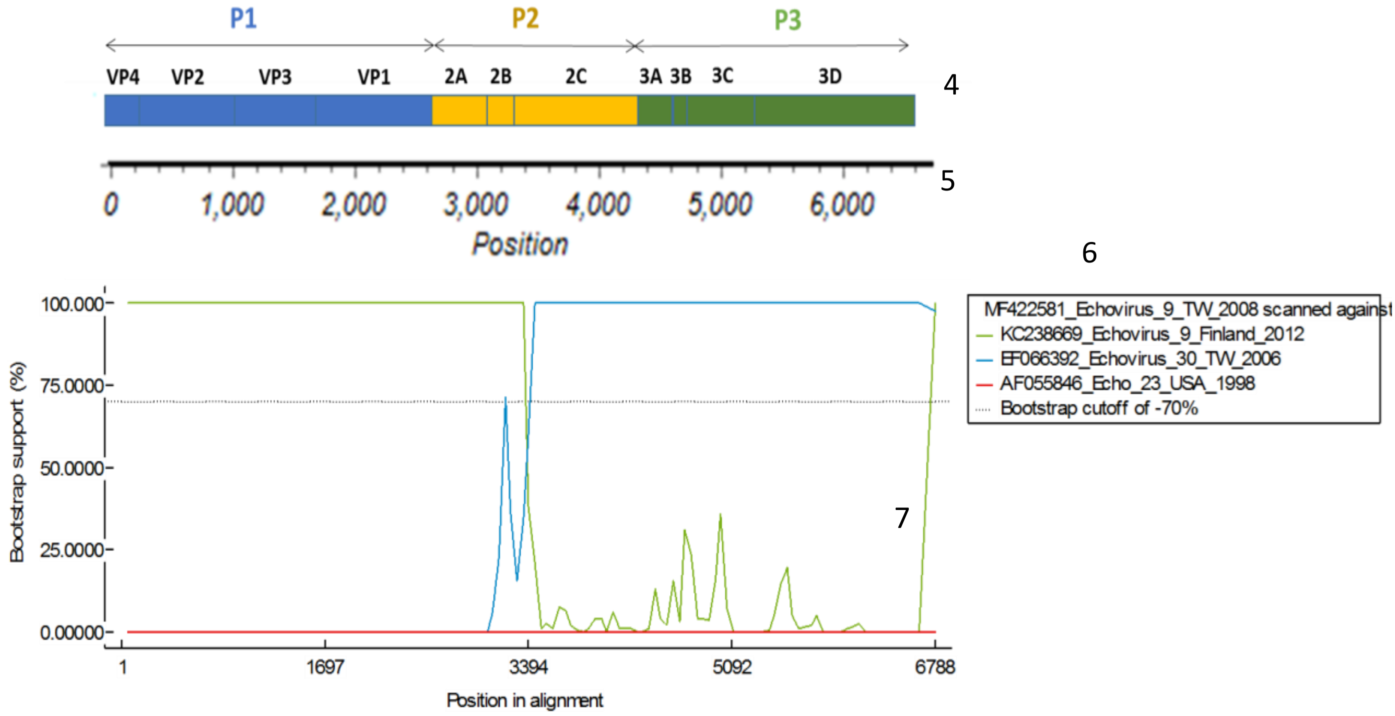

Supplement: Supplementary file 4 — Recombination analysis of MF422581_E9_TW_2008 using the RDP 4.0 program. The analyses were conducted via RDP 4.0 using the manual Bootscan method. Windows200 nt, step 20 nt. (PDF 194 kb) [file 12929_2019_541_MOESM4_ESM.pdf]
